# Anticipatory and consummatory pleasure in avoidant/restrictive food intake disorder

**DOI:** 10.1186/s40337-023-00921-w

**Published:** 2023-11-10

**Authors:** Sarah C. Dolan, P. Evelyna Kambanis, Casey M. Stern, Kendra R. Becker, Lauren Breithaupt, Julia Gydus, Sarah Smith, Madhusmita Misra, Nadia Micali, Elizabeth A. Lawson, Kamryn T. Eddy, Jennifer J. Thomas

**Affiliations:** 1 Department of Psychology, Hofstra University, Hempstead, NY USA; 2 Eating Disorders Clinical and Research Program, Massachusetts General Hospital, 2 Longfellow Place, Suite 200, Boston, MA 02114 USA; 3 Department of Psychiatry, Harvard Medical School, Boston, MA USA; 4 Department of Psychiatry, Athinoula A. Martinos Center for Biomedical Imaging, Charlestown, MA USA; 5 Neuroendocrine Unit, Massachusetts General Hospital, Boston, MA USA; 6 Department of Psychiatry, University of Geneva, Geneva, Switzerland; 7 Department of Pediatrics, Gynecology and Obstetrics, University of Geneva, Geneva, Switzerland; 8 Eating Disorders Research Unit, Mental Health Center Ballerup, Ballerup, Denmark

**Keywords:** Avoidant/restrictive food intake disorder, ARFID, Feeding and eating disorders, Pleasure, Anhedonia, Anticipatory pleasure, Consummatory pleasure, Sensory sensitivity, Fear of aversive consequences, Lack of interest in eating, Depression

## Abstract

**Background:**

Recent research suggests that individuals with eating disorders (EDs) report elevated anhedonia, or loss of pleasure. Although individuals with avoidant/restrictive food intake disorder (ARFID) often express that they do not look forward to eating, it is unclear whether they experience lower pleasure than those without EDs. Thus, identifying whether individuals with ARFID experience anhedonia may yield important insights that inform clinical conceptualization and treatment.

**Methods:**

A sample of 71 participants ages 10–23 with full and subthreshold ARFID and 33 healthy controls (HCs) completed the Pica, ARFID, and Rumination Disorder Interview, a diagnostic interview to assess ARFID profile severity (lack of interest in food, sensory sensitivity, fear of aversive consequences) and the Temporal Experience of Pleasure Scale (TEPS), a self-report measure of consummatory and anticipatory pleasure. Statistical analyses were performed using the full TEPS and also the TEPS with food-related items removed.

**Results:**

The ARFID group reported significantly lower anticipatory and consummatory pleasure compared to HCs, but these differences were no longer significant after controlling for depression, nor after removing food items from the TEPS. Within the ARFID sample, greater ARFID severity was associated with lower anticipatory pleasure across analyses, and greater endorsement of the lack of interest in food profile was related to lower anticipatory pleasure. ARFID severity was also associated with lower consummatory pleasure using the full TEPS, but this relationship was no longer significant with food items removed.

**Conclusions:**

These results provide initial evidence for lower pleasure before potentially pleasurable events in individuals with more severe ARFID, particularly those with the lack of interest phenotype. Our findings also suggest that depression is likely to contribute low pleasure in this population. Future research should seek to further characterize how dimensions of pleasure relate to the maintenance and treatment of ARFID symptoms.

## Abstract (plain language summary)

Individuals with eating disorders often report elevated anhedonia, or an inability to experience pleasure. Past research on pleasure in eating disorders has focused primarily on individuals with anorexia nervosa and bulimia nervosa, and it is unclear whether people with other eating disorders also experience lower pleasure than healthy individuals. In the current study, we measured anticipatory pleasure (looking forward to something enjoyable) and consummatory pleasure (enjoying a pleasant stimulus) in a sample with avoidant/restrictive food intake disorder (ARFID) and healthy controls. We also repeated our analyses after removing food-related items from the scale assessing pleasure. The ARFID group scored lower on both dimensions of pleasure than controls, but this difference was primarily due to greater depression symptoms and the presence of food-related items in the pleasure questionnaire. Within the ARFID sample, individuals with more severe ARFID reported less anticipatory pleasure, even after removing questions about enjoyment of food. Lower anticipatory pleasure was especially characteristic of the lack of interest in eating phenotype of ARFID. These results suggest that ARFID severity, lack of interest in eating, and depression contribute to low pleasure in this population.

## Introduction

Anhedonia, or the loss of pleasure and low reactivity to pleasurable stimuli [1], is observed across a wide range of psychiatric disorders [2, 3] and is associated with decreased response to psychiatric medications [4, 5] and longer time to remission from depressive episodes [6]. Research suggests that individuals with eating disorders (EDs) report lower overall pleasure compared to healthy controls [7], but existing literature has primarily examined pleasure and anhedonia in individuals with EDs characterized by weight and shape concerns, such as anorexia nervosa (AN) and bulimia nervosa (BN) [8–10], rather than the full range of eating pathology.

Individuals with avoidant/restrictive food intake disorder (ARFID), a disorder characterized by restricting the types or amount of food eaten without the goal of changing one’s weight or body shape, often express that they do not look forward to or enjoy eating [11]. The dimensional model of ARFID posits that individuals demonstrate symptoms consistent with three profiles (across continua of severity), including sensory sensitivity (avoiding foods because of aversion to taste, texture, or smell), lack of interest (avoiding eating sufficient food because of low appetite or low interest in food), and/or fear of aversive consequences (avoiding foods because of fear of choking, vomiting, or other adverse outcome) [11]. However, it is unclear whether a lack of interest or low enjoyment of food, which is considered a primary reward [12], generalizes to other potentially pleasurable stimuli. The study of anhedonia and pleasure in other EDs, particularly AN, has led to the development of reward-related theoretical models [13, 14] that have informed research efforts related to the role of reward in EDs and development of novel interventions that target low pleasure through increasing positive affect [15]. Therefore, investigating whether individuals with ARFID endorse low pleasure overall may lead to improved clinical conceptualization and treatment of this disorder.

The majority of research on pleasure has examined this construct in samples with AN, BN, and binge-eating disorder and suggests that, overall, individuals with EDs experience less pleasure [8, 9, 16, 17] and greater social anhedonia (low enjoyment of social situations) compared to controls [10, 18]. Furthermore, anhedonia persists after weight restoration in AN [8], suggesting that it may be linked to underlying psychopathology rather than a short-term consequence of malnutrition. Early findings also suggest that anhedonia is linked with ED severity, such that individuals with more severe ED symptoms report greater anhedonia [8, 10, 16]. To date, only two published studies have examined anhedonia in ARFID. One study found that individuals with ARFID reported less anhedonia (i.e., greater pleasure) than participants with AN, BN, binge-eating disorder, and other specified feeding or eating disorder [19], and another found no differences in pleasure across ED diagnoses [20]. Importantly, these studies did not test whether individuals with ARFID reported different levels of anhedonia compared to healthy control participants. Furthermore, specific ARFID profiles were not explored, so it is also important to determine whether each symptom profile differentially relates to anhedonia to further personalize interventions for ARFID.

Although existing research indicates that those with EDs experience general anhedonia, previous studies have largely studied pleasure as a unidimensional construct rather than incorporating multidimensional models of pleasure informed by advances in neuroscience [21, 22]. Research in other clinical and community samples suggests that pleasure can be separated into anticipatory (i.e., looking forward to future rewards/pleasurable stimuli) and consummatory (i.e., enjoying current rewards) subcomponents that are associated with distinct behavioral and neurobiological indices of reward motivation and enjoyment, respectively [23, 24]. Preliminary research suggests that among individuals with EDs characterized by weight and shape concerns, anticipatory and consummatory pleasure differentially relate to ED symptoms. For instance, one study found that greater anticipatory, but not consummatory, pleasure was associated with increased frequency of binge eating [25]. It is important to continue identifying how different components of pleasure play a role in other ED presentations.

## Aims and Hypotheses

### Hypothesis 1 (full sample)

In the current study, we aimed to test whether individuals with full and subthreshold ARFID [26] would differ from healthy control participants on a self-report measure of anticipatory and consummatory pleasure. Consistent with meta-analytic findings showing decreased pleasure in other ED samples with large effects [7], we hypothesized that the ARFID group would report lower pleasure in both domains.

### Hypothesis 2 (ARFID sample)

Within the ARFID sample, we also examined the relationship between ARFID symptom severity, depression, body mass index (BMI), age, and pleasure. Based on findings indicating that increased ED severity is related to greater anhedonia [8, 10, 16], we predicted that those with more severe ARFID symptoms would report lower anticipatory and consummatory pleasure, and this relationship would remain significant when controlling for depression and BMI, both of which have been associated with anhedonia in previous studies [27, 28].

### Exploratory analyses (ARFID sample)

We also conducted an exploratory analysis within the ARFID sample testing whether the three ARFID profiles (lack of interest in food, sensory sensitivity, and fear of aversive consequences) differentially related to anticipatory or consummatory pleasure. We did not have any a priori hypotheses for these analyses due to their exploratory nature.

### Food-related scale items

The TEPS has five items assessing anticipatory and consummatory pleasure related to food and drink. Because eating or feeding disturbance is a primary clinical feature of ARFID [1], we completed all analyses using both the full TEPS subscales, as well as the TEPS subscales with food-related items (i.e., items 3, 7, 8, 14, and 17) omitted.

## Method

### Participants

Participants were 71 individuals ages 10–23 years with ARFID and 33 age- and sex-matched healthy control (HCs) who completed clinical interviews and self-report questionnaires as part of a larger longitudinal study of ARFID (R01MH108595) in which we used structured interviews, hormone assays, and neuroimaging to characterize ARFID. As such, we wanted to capture children, adolescents, and adults, as ARFID occurs across the lifespan, but we also wanted to ensure children were old enough that they could fully participate in the interview and self-report measures. Using the World Health Organization’s definition of adolescence as ages 10 to 19 [29], 52 (73.2%) of participants in the ARFID group and 22 (66.7%) of HC participants were adolescents, and the remainder of participants were categorized as adults. Participants were recruited through local pediatric and adolescent medicine practices, eating disorder clinics, Rally (a Mass General Brigham recruitment platform), community advertisements, clinical research websites, flyers at local schools and colleges, and social media. Participants with ARFID were included if they met diagnostic criteria for full or subthreshold ARFID as assessed by clinical interview (see below for clinical interviewing procedures). Because our sample was predominantly composed of participants with full threshold ARFID (*n* = 63, 88.7%), we combined the full and subthreshold groups in data analysis, which is consistent with other published literature on ARFID [30–32]. HCs were included if they had a body mass index (BMI) in the 15th–85th percentiles and did not meet criteria for any lifetime diagnosis of psychiatric illness. Exclusion criteria for all participants included active suicidality, ED diagnosis other than ARFID, intellectual disability (IQ < 70), current substance use disorder, lifetime psychosis, use of systemic hormones (e.g., oral contraceptive pill), any contraindications for MRI scanning, and any significant medical condition that may interfere with study participation. Participants in the ARFID and HC groups did not differ on any demographic variables, but the ARFID group reported significantly greater ARFID severity and depression and lower BMI than HCs (see Table 1).

**Table 1:** Clinical and demographic characteristics of the sample

Variable	M(*SD*) or n (%)		*t/*χ^2^	*p*
	ARFID (*n* = 71)	HC (*n* = 33)		
Age (years)	16.78 (3.75)	16.75 (4.27)	0.03	0.97
Sex			0.04	0.84
Female	35 (49.3)	17 (51.5)		
Male	36 (50.7)	16 (48.5)		
Race			6.01	0.11
Asian	1 (1.40)	3 (9.10)		
Black/African American	0 (0)	1 (3.0)		
White	67 (94.40)	25 (75.80)		
More than one race	3 (4.20)	2 (6.10)		
Not reported	0 (0)	2 (6.10)		
Ethnicity			< 0.001	0.99
Hispanic or Latinx	8 (11.30)	3 (9.10)		
Non-Hispanic/Latinx	63 (88.70)	28 (84.80)		
Not reported	0	2 (6.10)		
PARDI severity	2.33 (0.84)	0.21 (0.24)	19.5	< 0.001
PARDI sensory profile	1.75 (1.36)	0.03 (0.10)	10.5	< 0.001
PARDI fear profile	0.43 (0.81)	0.17 (0.34)	4.40	< 0.001
PARDI lack of interest profile	2.02 (1.62)	0 (0)	9.11	< 0.001
Anticipatory pleasure	3.72 (0.92)	4.16 (0.88)	− 2.30	0.02
Consummatory pleasure	3.86 (1.01)	4.57 (1.03)	− 3.33	0.001
Depression *T*-score	53.07 (10.48)	42.73 (6.68)	6.05	< 0.001
BMI z-score	− 0.50 (1.62)	0.19 (0.69)	3.07	0.003

*ARFID* avoidant/restrictive food intake disorder, *BMI* body mass index, *HC* healthy control, *PARDI* pica, ARFID and rumination disorder interview, *TEPS* temporal experience of pleasure scale. BMI *z*-scores were calculated using CDC weight-for-age reference populations. The ARFID group included participants with both full and subthreshold ARFID

## Measures

### Clinical interviews

We used the Kiddie Schedule for Affective Disorders and Schizophrenia—Present and Lifetime Version (KSADS-PL) [33] to assess current and lifetime psychiatric comorbidities and the Eating Disorder Assessment for *DSM-5* (EDA-5) [34] to screen for the presence of ARFID symptoms and other exclusionary ED diagnoses. Although the KSADS-PL was developed for use in children ages 6–17 [33], it has been used with participants up to age 25 [35, 36]; therefore, we used this interview for all participants to maintain consistency.

Once initial eligibility was confirmed, the Pica, ARFID, and Rumination Disorder Interview (PARDI) [37] was used to confirm ARFID diagnosis (full or subthreshold) and symptom severity. Participants were diagnosed with full ARFID if they met one or more of the four components of diagnostic Criterion A (weight loss, nutritional deficiency, dependence on nutrition supplements, or marked interference with psychosocial functioning) at the severity level required on the PARDI; alongside Criteria B (eating disturbance is not explained by lack of available food and/or cultural practice), C (eating disturbance is not related to weight/shape concerns), and D (eating disturbance is not explained by another medical or psychiatric disorder). Participants were diagnosed with subthreshold ARFID if they reported clinically significant avoidant or restrictive eating that met Criteria B, C, and D but did not report symptoms that met the PARDI severity threshold for Criterion A (e.g., scoring three out of six on psychosocial impairment, when the threshold for diagnosis full threshold ARFID is four out of six). Items are rated on a scale from 0 to 6, with higher scores indicating greater severity. Participants’ ARFID severity score was computed by taking the average of all items assessing the level of functional impairment and medical severity due to ARFID symptoms. The PARDI also includes items that assess three ARFID profiles: lack of interest in eating/food, sensory sensitivity, and fear of aversive consequences. Scores for each ARFID profile were computed by taking the average of the items used to measure that profile, such that individuals could demonstrate symptoms of any combination of profiles rather than exhibiting one primary presentation. Using previously validated clinical cutoffs for each profile [38],^1^ 45 participants (63.4%) endorsed features consistent with a lack of interest profile, 54 participants (76.1%) endorsed features consistent with a sensory sensitivity profile, and 17 participants (23.9%) endorsed features consistent with a fear of aversive consequences profile. In a larger validation study of the PARDI, which included a subset of the current sample, interrater reliability was good, κ = 0.75 [37].

### Pleasure

The Temporal Experiences of Pleasure Scale (TEPS) [39] is an 18-item self-report measure with a subscale that measures anticipatory pleasure (i.e., looking forward to pleasurable events) and a subscale that measures consummatory pleasure (i.e., enjoying a pleasant event as it occurs). Items are rated on a scale from 1 to 6 and then averaged for each subscale, with greater scores indicating higher pleasure. For example, “*When something exciting is coming up in my life, I really look forward to it*” is an item assessing anticipatory pleasure, and “*I enjoy taking a deep breath of fresh air when I walk outside*” is an item assessing consummatory pleasure. While not previously used in a sample with ARFID, the TEPS has been used in samples with other ED diagnoses [25] and other clinical and community samples [40, 41]. We conducted statistical analyses using both the full measure and also with food-related items omitted (e.g., *"When ordering something off the menu, I imagine how good it will taste" *was omitted from the anticipatory subscale, and *"A hot cup of coffee or tea on a cold morning is very satisfying to me"* was omitted from the consummatory subscale). The TEPS anticipatory pleasure subscale demonstrated good internal consistency in this sample, α = 0.84, and the consummatory pleasure subscale demonstrated acceptable internal consistency, α = 0.73. When items related to food were omitted, the anticipatory pleasure subscale demonstrated acceptable internal consistency, α = 0.77, but the consummatory pleasure subscale demonstrated poor internal consistency, α = 0.52.

### Depression

Participants ages 18 years and older completed the Beck Depression Inventory (BDI-II) [42], a widely used self-report measure that assesses the severity of depression symptoms. Participants under age 18 years completed the Children’s Depression Inventory (CDI) [43], a similar self-report measure developed for children and adolescents. To combine scores on these measures for all participants, each participant’s raw score on their depression measure was converted to a *T*-score using community norms included in the administration manual for each measure, with higher *T*-scores indicating more severe depressive symptoms. The BDI had excellent internal consistency, α = 0.94, and the CDI had good internal consistency, α = 0.88.

### BMI

Study staff measured participants’ height and weight using a stadiometer and calibrated scale. Height was measured three times and study staff recorded the average of these measurements. For adult participants, we computed BMI; for participants under age 18 years, we calculated the percent expected body weight (EBW). To standardize the sample based on age and sex, we calculated the BMI *z*-score based on Center for Disease Control reference populations, which range from ages two to 20 [44]. For participants whose age was outside this range (i.e., age 21–23), we calculated their BMI *z*-score using a reference age of 20.

### Statistical analyses

#### ***Hypothesis 1***

To test the hypothesis that participants with full and subthreshold ARFID would report lower anticipatory and consummatory pleasure compared to the healthy control group, we used two sample *t*-tests for each TEPS subscale and followed up significant tests with an ANCOVA with depression symptoms and BMI z-score entered as covariates. Both dependent variables had homogeneity of variance across groups as assessed by Levene’s test, *p* > 0.05.

#### ***Hypothesis 2***

To test the hypothesis that greater ARFID severity would be associated with lower anticipatory and consummatory pleasure, we first computed Pearson correlation coefficients between ARFID severity, TEPS subscales, BMI, and depression. We followed up significant correlations with two linear regression analyses within the ARFID sample with anticipatory and consummatory pleasure as the dependent variables, respectively. The PARDI ARFID severity score was the independent variable; depression and BMI were entered as covariates.

#### Exploratory analyses

For our exploratory analyses examining the relationships between ARFID profiles and pleasure within the ARFID sample, we first computed Pearson correlation coefficients between ARFID profiles and TEPS subscales. We followed up significant correlations with a linear regression analysis with anticipatory pleasure as the dependent variable and ARFID profile severity scores (sensory sensitivity, fear of aversive consequences, lack of interest in food/eating) as the independent variables. We entered depression and BMI z-score as covariates.

## Results

### Hypothesis 1: group differences in anticipatory and consummatory pleasure

#### Full TEPS subscales

Consistent with our hypothesis, the ARFID group endorsed significantly lower anticipatory pleasure, *t*(102) = − 2.30, *p* = 0.02, *d* = 0.49, and consummatory pleasure, *t*(102) = − 3.33, *p* = 0.001, *d* = 0.70, than the healthy control group.

However, in contrast to our predictions, group differences in pleasure did not remain significant when controlling for depression and BMI. The overall model assessing group differences in anticipatory pleasure was significant, *F*(3, 99) = 6.41, *p* < 0.001, but depression was the only variable with a significant effect on anticipatory pleasure, *F*(1, 99) = 13.34, η^2^ = 0.11, *p* < 0.001. In the ANCOVA assessing group differences in consummatory pleasure, the overall model was significant, *F*(3, 99) = 5.81, *p* = 0.001 and depression was the only variable with a significant effect *F*(1, 99) = 6.10, η^2^ = 0.05, *p* = 0.02 (see Table 2 for estimated marginal means of anticipatory and consummatory pleasure for each group). The results from these models suggest that individuals with greater depression symptoms experience decreased anticipatory and consummatory pleasure, regardless of diagnostic status and BMI.

**Table 2:** Estimated marginal means of anticipatory and consummatory pleasure when controlling for age, depression, and body mass index

All items	ARFID	HC
Anticipatory pleasure (95% CI)	3.83 (3.62–4.05)	3.93 (3.59–4.26)
Consummatory pleasure (95% CI)	3.94 (3.70–4.18)	4.41 (4.04–4.78)
*Without food items*		
Anticipatory pleasure (95% CI)	3.79 (3.53–4.04)	3.73 (3.34–4.11)
Consummatory pleasure (95% CI)	4.08 (3.87–4.29)	4.02 (3.70–4.35)

*ARFID* avoidant/restrictive food intake disorder, *CI* confidence interval, *HC* healthy control

#### TEPS subscales without food items

In contrast with our hypothesis, there were no group differences in anticipatory pleasure, *t*(102) = − 1.62, *p* = 0.11, and consummatory pleasure, *t*(102) = − 1.13, *p* = 0.26, when food items were omitted from the TEPS. Because the ARFID and HC groups did not differ significantly on either TEPS subscale, we did not perform follow-up analyses assessing for the effects of depression and BMI.

### Hypothesis 2: relationship between ARFID severity and pleasure

#### Full TEPS subscales

The correlation coefficients between ARFID severity, anticipatory pleasure, and consummatory pleasure are presented in Table 3. ARFID severity (as measured by the PARDI severity scale) was positively correlated with depression, *r* = 0.43, *p* ≤ 0.001, and negatively associated with anticipatory pleasure, *r* = − 0.39, *p* ≤ 0.001, and consummatory pleasure, *r* = − 0.32, *p* = 0.01, indicating that individuals with greater ARFID severity experience greater depression *and* lower pleasure. Age was not significantly related to anticipatory pleasure, *r* = − 0.09, *p* = 0.47, or consummatory pleasure, *r* = 0.19, *p* = 0.11.

**Table 3:** Correlations between study variables in the ARFID sample

	PARDI	BMI	Depression	Consummatory pleasure	Anticipatory pleasure	Fear	Interest
Sensory	0.53**	0.05	0.22	− 0.05	− 0.26*	0.18	0.33**
Interest	0.41**	− 0.30*	0.31*	0.01	− 0.31*	0.34**	
Fear	0.04	− 0.22	− 0.06	0.20	0.06		
Anticipatory pleasure	− 0.39**	− 0.06	− 0.41**	0.66**			
Consummatory pleasure	− 0.32**	0.03	− 0.23				
Depression	0.43**	0.14					
BMI	0.08						

Items in bold are statistically significant

*ARFID avoidant/restrictive food intake disorder, BMI* body mass index, *PARDI* pica, ARFID, and rumination disorder interview

*p < 0.05

**p < 0.01

The full results of regression models used to assess whether correlations remained significant when controlling for relevant covariates are presented in Table 5. The overall model testing the relationships between anticipatory pleasure, ARFID severity, depression, and BMI was significant, *R*^*2*^ = 0.19, *F*(3, 65) = 6.22, *p* < 0.001. Consistent with our hypothesis, ARFID severity was negatively related to anticipatory pleasure, *B* = − 0.30, *p* = 0.03. Depression, but not BMI, was significantly negatively associated with anticipatory pleasure, *B* = − 0.02, *p* = 0.03. These results indicate that, among individuals with ARFID, those with greater ARFID severity *and* greater depression severity report lower anticipatory pleasure.

The overall model testing relationships between consummatory pleasure, ARFID severity, depression, and BMI was also significant, *R*^*2*^ = 0.07, *F*(3, 65) = 2.80, *p* = 0.05. ARFID severity was the only variable in the model that was significantly related to consummatory pleasure, *B* = − 0.33, *p* = 0.04, which suggests individuals with more severe ARFID symptoms report lower consummatory pleasure independent of depression symptoms and BMI.

#### TEPS subscales without food items

The correlation coefficients between ARFID severity, anticipatory pleasure, and consummatory pleasure are presented in Table 4. ARFID severity was negatively associated with anticipatory pleasure, *r* = − 0.40, *p* ≤ 0.001, but its relationship with consummatory pleasure was no longer significant after omitting food-related TEPS items, *r* = − 0.16, *p* = 0.18. Similar to the results of analyses with the full TEPS subscales, age was not significantly related to anticipatory pleasure, *r* = 0.01, *p* = 0.93, or consummatory pleasure, *r* = 0.07, *p* = 0.59.

**Table 4:** Correlations between study variables and anticipatory and consummatory pleasure with food-related items omitted

	PARDI	Fear	Interest	Sensory	BMI	Depression
Anticipatory pleasure	− 0.40**	0.05	− 0.33**	− 0.21	0.05	− 0.36**
Consummatory pleasure	− 0.16	0.27*	0.03	0.03	− 0.11	− 0.30*

Items in bold are statistically significant

*BMI* body mass index, *PARDI* pica, ARFID, and rumination disorder interview

*p < 0.05

**p < 0.01

The full results of regression models used to assess whether correlations remained significant when controlling for relevant covariates are presented in Table 5. When omitting food-related items, the overall model testing the relationships between anticipatory pleasure, ARFID severity, depression, and BMI remained significant, *R*^*2*^ = 0.17, *F*(3, 65) = 5.69, *p* = 0.002. ARFID severity remained negatively related to anticipatory pleasure, *B* = − 0.36, *p* = 0.02, but depression was no longer significantly associated with anticipatory pleasure, *B* = − 0.02, *p* = 0.06.

**Table 5:** Regression analyses exploring associations between pleasure and ARFID severity within the ARFID sample

DV: anticipatory pleasure	Adj. *R*^2^	*B*	*SE*	*t*	*η*^*2*^	*p*
Full model: *F*(3, 65) = 6.22, *p* < 0.001	0.19					
Constant		5.69	0.53	10.70		< 0.001
ARFID severity		− 0.30	0.13	− 20.27	0.16	0.03
Depression		− 0.02	0.01	20.24	0.05	0.03
BMI		− 0.02	0.06	− 0.39	< 0.01	0.70
*DV: anticipatory pleasure (without food items)*						
Full model: *F*(3, 65) = 5.69, *p* = 0.002	0.17					
Constant		5.78	0.61	9.55		< 0.001
ARFID severity		− 0.36	0.15	− 2.38	0.16	0.02
Depression		− 0.02	0.01	− 1.91	0.04	0.06
BMI		0.04	0.07	0.57	0.003	0.57
*DV: consummatory pleasure*						
Full model: *F*(3, 65) = 2.80, *p* = 0.05	0.07					
Constant		5.20	0.63	8.29		< 0.001
ARFID severity		− 0.33	0.16	− 2.09	0.20	0.04
Depression		− 0.01	0.01	− 0.83	0.01	0.41
BMI		0.02	0.07	0.21	< 0.01	0.83
*DV: consummatory pleasure (without food items)*						
Full model: *F*(3, 65) = 2.21 *p* = 0.09	0.05					
Constant		5.29	0.57	9.21		< 0.001
ARFID severity		− 0.06	0.14	− 0.43	0.03	0.66
Depression		− 0.02	0.01	− 1.91	0.06	0.06
BMI		− 0.04	0.07	0.68	0.01	0.49

*ARFID* avoidant/restrictive food intake disorder, *BMI* body mass index

In contrast to the model with the all TEPS items, the overall model testing relationships between consummatory pleasure, ARFID severity, depression, and BMI was not significant, *R*^*2*^ = 0.05, *F*(3, 65) = 2.21, *p* = 0.09.

### Exploratory analyses of relationships between pleasure and ARFID profiles

#### Full TEPS subscales

Scores on the sensory sensitivity profile were negatively associated with anticipatory pleasure, *r* = − 0.26, *p* = 0.03, as were scores on the lack of interest in food profile, *r* = − 0.31, *p* = 0.01; consummatory pleasure was not associated with any ARFID profile (see Table 3). These results suggest that, while individuals with greater sensory sensitivity and less interest in food may report lower anticipatory pleasure, none of the ARFID profiles were associated with level of consummatory pleasure.

The full results of a follow-up regression model controlling for depression and BMI are presented in Table 6. The overall model assessing relationships between ARFID profiles, depression, BMI, and anticipatory pleasure was significant, *R*^*2*^ = 0.17, *F*(5, 63) = 3.87, *p* = 0.04. Although sensory sensitivity and lack of interest in food were negatively related to anticipatory pleasure in correlation analyses, depression was the only variable in this model that had a significant relationship with anticipatory pleasure (*B* = − 0.03, *p* = 0.02). These results indicate that, among individuals with ARFID, greater depressive symptoms were associated with decreased anticipatory pleasure, regardless of specific ARFID profile.

**Table 6:** Regression analyses exploring associations between anticipatory pleasure and ARFID profiles within the ARFID sample

DV: anticipatory pleasure	Adj. *R*^2^	*B*	*SE*	*t*	*η*^*2*^	*p*
Full model: *F*(5, 63) = 3.87, *p* = 0.004	0.17					
Constant		5.38	0.54	9.85		< 0.001
Sensory sensitivity		− 0.09	0.08	− 1.09	0.03	0.28
Lack of interest in food		− 0.14	0.08	− 1.80	0.13	0.07
Fear of aversive consequences		0.15	0.14	1.09	< 0.01	0.28
BMI		− 0.03	0.07	− 0.47	< 0.01	0.64
Depression		− 0.03	0.01	− 2.33	0.07	0.02
*DV: anticipatory pleasure (without food items)*						
Full model: *F*(5, 63) = 3.41, *p* = 0.01	0.15					
Constant		5.39	0.63	8.61		< 0.001
Sensory sensitivity		− 0.06	0.09	− 0.69	0.01	0.49
Lack of interest in food		− 0.18	0.09	− 2.02	0.15	0.05
Fear of aversive consequences		0.19	0.15	1.22	0.002	0.22
BMI		0.03	0.08	0.37	0.05	0.71
Depression		− 0.03	0.08	− 2.04	0.002	0.05
*DV: consummatory pleasure (without food items)*						
Full model: *F*(5, 63) = 2.27, *p* = 0.06	0.09					
Constant		5.13	0.58	8.93		< 0.001
Sensory sensitivity		0.04	0.08	0.42		0.68
Lack of interest in food		0.01	0.08	0.10		0.92
Fear of aversive consequences		0.26	0.14	1.87		0.07
BMI		− 0.01	0.07	− 0.17		0.86
Depression		− 0.03	0.01	− 2.27		0.03

*ARFID* avoidant/restrictive food intake disorder, *BMI* body mass index

#### TEPS subscales without food items

Correlations between TEPS subscales and ARFID profiles are presented in Table 4. When food items were removed, the lack of interest in food profile remained negatively correlated with consummatory pleasure, *r* = − 0.33, *p* = 0.005, but the sensory sensitivity profile was no longer significantly associated with anticipatory pleasure. In contrast to analyses with all TEPS items, the fear of aversive consequences profile was positively correlated with consummatory pleasure when food items were removed, *r* = 0.27, *p* = 0.02. These results suggest that, when pleasure related to food is not assessed, individuals with the lack of interest ARFID profile experience decreased anticipatory pleasure and individuals with the fear of aversive consequences profile experience greater consummatory pleasure.

Because both anticipatory and consummatory pleasure were correlated with at least one subscale when food items were removed, we completed follow-up regression models for both TEPS subscales. The full results of regression models controlling for depression and BMI are presented in Table 6. The overall model assessing relationships between ARFID profiles, depression, BMI, and anticipatory pleasure remained significant, *R*^*2*^ = 0.15, *F*(5, 63) = 3.41, *p* = 0.01. Depression remained significantly negatively related to anticipatory pleasure, *B* = − 0.03, *p* = 0.05; in contrast with the model using all TEPS items, lack of interest score was significantly negatively associated with anticipatory pleasure, *B* = − 0.17, *p* = 0.05, suggesting that the relationship between these two constructs is strengthened when food-related pleasure is not measured.

The model assessing relationships between ARFID profiles, depression, BMI, and consummatory pleasure was not significant, *R*^*2*^ = 0.09, *F*(5, 63) = 2.27, *p* = 0.06, but depression was negatively related to consummatory pleasure, *B* = − 0.03, *p* = 0.03, indicating that depressive symptoms, rather than specific ARFID presentations, may contribute to the variance in consummatory pleasure within this population.

## Discussion

The current study is the first to compare self-reported pleasure in participants with ARFID and healthy controls. In contrast to the majority of prior research on pleasure and anhedonia in EDs, which measure pleasure as a unidimensional construct [7], we assessed two subcomponents: anticipatory and consummatory pleasure. Individuals with ARFID in our sample endorsed lower anticipatory and consummatory pleasure compared to HCs, but these differences were no longer significant when controlling for depression and omitting scale items that assessed pleasure related to food. However, the negative relationship between ARFID severity and anticipatory pleasure remained consistent across analyses. This pattern of results indicates that participants with more severe ARFID may be more likely to have deficits in anticipatory pleasure, which is consistent with previous research suggesting that anticipatory pleasure may be more relevant than consummatory pleasure to eating pathology across ED diagnoses [25].

Within the ARFID sample, greater ARFID and depression severity both related to lower anticipatory pleasure, and this finding remained after omitting food items, indicating that individuals with more severe ARFID and/or comorbid depression experience low anticipatory pleasure beyond disorder-relevant stimuli. When using all available TEPS items, ARFID severity was negatively associated with consummatory pleasure over and above depression, but this finding was not significant after removing food items, which suggests that items assessing enjoyment of food may have been a confounding variable in this population.

In exploratory analyses testing relationships between ARFID profiles and pleasure, lower anticipatory pleasure was correlated with greater severity of the lack of interest profile, regardless of whether food items were included or removed. This finding points to the potential relevance of anticipatory pleasure in some ARFID symptoms and suggests that individuals who express that they do not look forward to eating may also experience low anticipatory pleasure more broadly. Although sensory sensitivity was negatively correlated with anticipatory pleasure when using the full TEPS subscale, this relationship was no longer significant after omitting food items, suggesting that the initial correlation was due to low ratings on food items, but anticipatory and consummatory pleasure related to other stimuli remain intact in individuals with this presentation. No ARFID profile uniquely predicted consummatory pleasure when controlling for depression in any analyses, which indicates that any observed consummatory anhedonia in this population may be attributed to comorbid depression.

Previous research on EDs characterized by weight and shape concerns (e.g., AN, BN) demonstrates that individuals with EDs report lower overall pleasure compared to controls [8, 18, 45]. Our findings add to the existing body of literature on anhedonia and pleasure in EDs by indicating that deficits in pleasure in ARFID are particularly relevant for individuals with more severe ARFID symptoms and those with lack of interest in food. Notably, a number of analyses produced different results when we omitted TEPS items that were related to food. Although many self-report measures of anhedonia and pleasure include items that assess enjoyment of food and drink [39, 46, 47], most studies using these measures in samples with EDs do not report results of analyses without those items; future research should determine whether samples with other ED diagnoses report different levels of pleasure when food-related scale items are removed.

While our study provided novel insights into whether individuals with ARFID experience low pleasure, it also had limitations. First, we used a mixed child and adult sample (ages 10–23 years), and the TEPS has not previously been validated for use in a child sample. However, the TEPS has been used in adolescent samples with participants as young as age 13 with acceptable to good internal consistency [48, 49]. Of note, the consummatory pleasure subscale of the TEPS demonstrated low internal consistency once food-related items were removed, which may have limited our power to detect relationships. Furthermore, our data were cross-sectional, so we cannot draw conclusions about whether low pleasure is a risk factor for developing ARFID, or whether ARFID symptoms cause individuals to experience less pleasure. Our study’s findings would also have been strengthened by using one of the well-validated behavioral reward processing tasks that are associated with self-reported anticipatory and/or consummatory pleasure, such as the Effort Expenditure for Reward Task [50] or a probabilistic reward task [51]. Our sample was also predominantly White and non-Hispanic, so it is unclear whether these findings would generalize to a sample with greater racial/ethnic diversity.

Limitations notwithstanding, the results of the current study not only provide important preliminary evidence of alterations in anticipatory pleasure in ARFID, but also hold potential implications for ARFID treatment. Importantly, individuals with greater ARFID symptom severity reported lower anticipatory pleasure, which indicates that, similar to other populations that report anhedonia, increasing pleasure may be a relevant intervention target [15, 52]. Of note, our findings highlight a potential mechanism through which food-based exposures in ARFID could facilitate symptom change. Specifically, if consummatory pleasure in ARFID remains intact, the act of eating during food exposure might ultimately be more pleasurable than anticipated, creating an expectancy violation that challenges patients’ low anticipatory pleasure about eating. This interpretation would be consistent with an inhibitory learning model of symptom change in ARFID [53]. Because anhedonia has been linked to poor treatment response in other clinical samples [5, 6], future research should investigate anticipatory and consummatory pleasure as potential mediators and/or moderators of symptom trajectory and treatment outcome in ARFID.

Overall, the results of the current study suggest that greater symptom severity and co-occurring depression symptoms both contribute to deficits in pleasure in individuals with ARFID. These findings emphasize the importance of future research on pleasure and anhedonia in the ARFID population, particularly as this construct relates to treatment implications and longitudinal course.

## Footnotes

1 Clinical cutoffs from this paper were used for the lack of interest profile and the sensory sensitivity profile. Because the referenced paper indicated that the cutoff for the fear of aversive consequences did not have adequate specificity, we used a lower cutoff based on the distribution of our sample.
